# Confocal Microscopy in Biopsy Proven Argyrosis

**DOI:** 10.1155/2013/875989

**Published:** 2013-07-18

**Authors:** Melis Palamar, Suzan Guven Yilmaz, Taner Akalin, Sait Egrilmez, Ayse Yagci

**Affiliations:** ^1^Department of Ophthalmology, Ege University Faculty of Medicine, 35100 Izmir, Turkey; ^2^Department of Pathology, Ege University Faculty of Medicine, 35100 Izmir, Turkey

## Abstract

*Purpose*. To evaluate the confocal microscopy findings of a 46-year-old male with bilateral biopsy proven argyrosis. *Materials and Methods*. Besides routine ophthalmologic examination, anterior segment photography and confocal microscopy with cornea Rostoch module attached to HRT II (Heidelberg Engineering GmbH, Heidelberg, Germany) were performed. *Findings*. Squamous metaplastic changes on conjunctival epithelium and intense highly reflective extracellular punctiform deposits in conjunctival substantia propria were detected. Corneal epithelium was normal. Highly reflective punctiform deposits starting from anterior to mid-stroma and increasing through Descemet's membrane were evident. Corneal endothelium could not be evaluated due to intense stromal deposits. *Conclusion*. Confocal microscopy not only supports diagnosis in ocular argyrosis, but also demonstrates the intensity of the deposition in these patients.

## 1. Introduction

Prolonged exposure to silver might cause irreversible pigmentation of the skin (argyria) and/or the eyes (argyrosis) [1]. Hands, eyes, and mucous membranes are affected in most of the patients, and discoloration of the ocular surface is the main ocular evidence [1–3]. A direct relationship was shown between the amount of discoloration and the length of time worked [1]. 

Confocal microscopy provides high-resolution, high-contrast in vivo images and is a powerful tool for studying the ultrastructure of the cell, its molecular components, and their functions. The Rostock cornea module is an option of the Heidelberg Retina Tomograph (HRT II, Version 3.0; Heidelberg Engineering GMBH, Dossenheim, Germany) introduced as an improvement over older confocal microscopes. The module consists of a monochrome laser radiation source which avoids chromatic aberrations and provides extremely sharp images and a high-powered lens that allows the operator to change the confocal plane within the cornea to capture images at different depths without losing sharpness [4].

The aim of this study is to demonstrate the location of conjunctival and corneal silver deposits by confocal microscopy and to evaluate the correlation between conjunctival histopathology and confocal microscopy findings in a case of occupational argyrosis. To the best of our knowledge this is the first biopsy proven argyrosis case to be evaluated by confocal microscopy. 

## 2. Report of a Case

**Figure 1:**
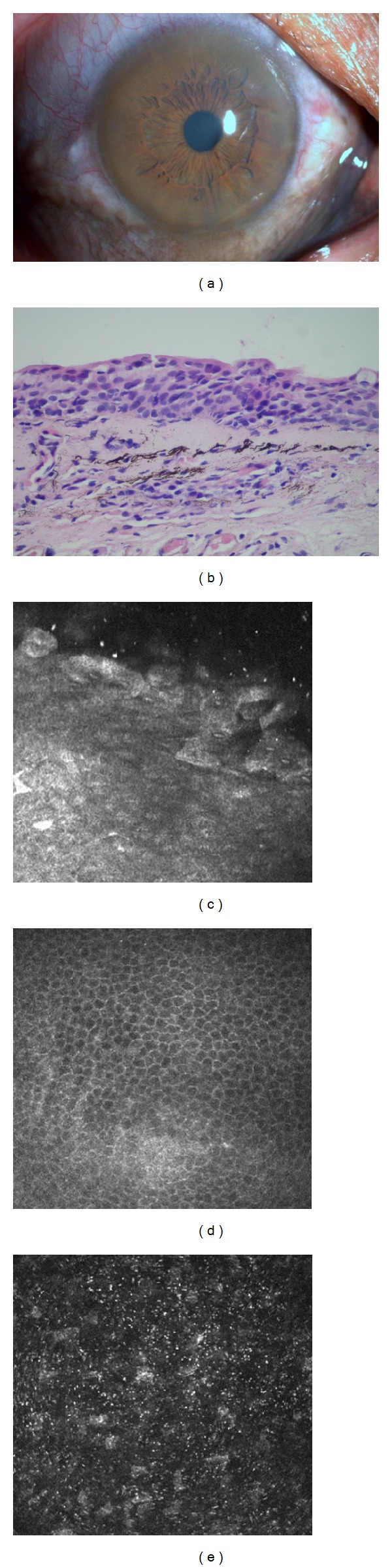
(a) Diffuse black-gray pigmentation in ocular surface in both eyes. (b) Histopathology revealed subepithelial extracellular silver particles in the substantia propria. (c) Squamous metaplastic changes on conjunctival epithelium and diffuse highly reflective extracellular punctiform deposits in conjunctival substantia propria. (d) Normal corneal epithelium on confocal microscopy. (e) Highly reflective punctiform deposits in the stroma demonstrated with confocal microscopy.

A 46-year-old long-standing silver worker who was diagnosed as ocular argyrosis 4 years earlier was evaluated with confocal microscopy. His visual acuity was 10/20 and intraocular pressures were normal bilaterally. The periocular skin and ocular surface displayed diffuse black-gray pigmentation in both eyes (Figure 1(a)). Patchy pigmentation was present in the corneal stroma and Descemet' membrane bilaterally. Fundus examination revealed no pigmentation. Histopathologic examination of the conjunctival incisional biopsy material revealed squamous metaplastic changes at the epithelium level and subepithelial extracellular silver particles in the substantia propria, which supported the diagnosis of argyrosis (Figure 1(b)).

On confocal microscopy, squamous metaplastic changes on conjunctival epithelium and diffuse highly reflective extracellular punctiform deposits in conjunctival substantia propria were detected bilaterally (Figure 1(c)). Corneal epithelium was normal, and highly reflective punctiform deposits starting from anterior- to mid-stroma and increasing through corneal endothelium were evident in both eyes (Figures 1(d) and 1(e)). Corneal endothelium could not be evaluated probably due to intense stromal deposits.

## 3. Comment

Gray discoloration of the conjunctiva and scattered gray opacities in the cornea are clinical ocular manifestations of argyrosis [1–3]. Our patient had generalized argyria—conjunctival, corneal pigmentation as well as skin, nail, and dental pigmentation resulting from occupational contact. 

 Confocal microscopy is a noninvasive tool for in vivo examination of diseases affecting the ocular surface. It provides images of histologic sections of tissue giving a better sense of the three-dimensional nature of the pathology [4]. The HRT is the only instrument that uses laser technology (laser diode 670 nm). The main advantage of this system is that scanning confocal chromatic aberration is eliminated from the laser light source rendering better-defined images [4]. Confocal microscopy examination of the cornea in our case revealed changes starting from anterior to mid-stroma through endothelium without epithelial involvement. The deposits appeared in a scattered fashion of hyperreflective dots as reported earlier [3]. The endothelium could not be visualized, probably due to highly reflective deposits increasing through deeper layers of the stroma. However, Sánchez-Pulgarín et al. [3] previously reported that the endothelium was normal in ocular argyrosis. Squamous metaplastic changes on conjunctival epithelium and diffuse highly reflective extracellular punctiform deposits in conjunctival substantia propria were detected on confocal microscopy as was demonstrated with histopathologic examination. Although we did not confirm the corneal deposits by biopsy, conjunctival confocal microscopy results supported conjunctival histopathology results which approved argyrosis. As discussed earlier by Sánchez-Pulgarín et al. [3] epithelial metaplasia of conjunctiva might correspond either to the silver deposits or to structural changes induced by the disease. 

In vortex keratopathy—either due to Fabry disease or due to amiodarone use—the corneal deposits are intracellular due to systemic retention [5]. However, in occupational argyrosis which does not interfere systemic circulation, the detected deposits are extracellular—contrary to vortex keratopathy [3]. 

Confocal microscopy is a noninvasive diagnostic tool in ocular argyrosis. This technique provides high quality images which display high correlation with histopathologic results. Therefore, in patients opposing surgical intervention, confocal microscopy can be a safe and a reliable approach for diagnosis.
